# An Empirical Study on V2X Enhanced Low-Cost GNSS Cooperative Positioning in Urban Environments †

**DOI:** 10.3390/s19235201

**Published:** 2019-11-27

**Authors:** Paul Schwarzbach, Albrecht Michler, Paula Tauscher, Oliver Michler

**Affiliations:** Chair of Transport Systems Information Technology, Institute of Traffic Telematics, TU Dresden, 01069 Dresden, Germany

**Keywords:** cooperative positioning, GNSS, V2X, low-cost GNSS, DGNSS, VANET, C-ITS, ITS-G5

## Abstract

High-precision and lane selective position estimation is of fundamental importance for prospective advanced driver assistance systems (ADAS) and automated driving functions, as well as for traffic information and management processes in intelligent transportation systems (ITS). User and vehicle positioning is usually based on Global Navigation Satellite System (GNSS), which, as stand-alone positioning, does not meet the necessary requirements in terms of accuracy. Furthermore, the rise of connected driving offers various possibilities to enhance GNSS positioning by applying cooperative positioning (CP) methods. Utilizing only low-cost sensors, especially in urban environments, GNSS CP faces several demanding challenges. Therefore, this contribution presents an empirical study on how Vehicle-to-Everything (V2X) technologies can aid GNSS position estimation in urban environments, with the focus being solely on positioning performance instead of multi-sensor data fusion. The performance of CP utilizing common positioning approaches as well as CP integration in state-of-the-art Vehicular Ad-hoc Networks (VANET) is displayed and discussed. Additionally, a measurement campaign, providing a representational foundation for validating multiple CP methods using only consumer level and low-cost GNSS receivers, as well as commercially available IEEE 802.11p V2X communication modules in a typical urban environment is presented. Evaluating the algorithm’s performance, it is shown that CP approaches are less accurate compared to single positioning in the given environment. In order to investigate error influences, a skyview modelling seeking to identify non-line-of-sight (NLoS) effects using a 3D building model was performed. We found the position estimates to be less accurate in areas which are affected by NLoS effects such as multipath reception. Due to covariance propagation, the accuracy of CP approaches is decreased, calling for strategies for multipath detection and mitigation. In summary, this contribution will provide insights on integration, implementation strategies and accuracy performances, as well as drawbacks for local area, low-cost GNSS CP in urban environments.

## 1. Introduction

Intelligent transportation systems (ITS) are a major factor in the future of multi-modal mobility systems. The interconnection and networking of vehicles, traffic management and infrastructure enhances road safety and traffic flow control, as well as consumer and infotainment applications. With the emergence of advanced driver assistance systems (ADAS), highly-automated (HAD), or even autonomous driving, vehicular communication is a necessary prerequisite as it extends the electronic horizon of connected vehicles, further augmenting environmental perception (cooperative sensing) and mutual movement prediction (cooperative maneuvering) [1]. Further on, highly accurate positioning of traffic participants is currently one of the greatest challenges in the field of ITS. It functions as an essential fundamental for both safety relevant comfort and informational applications as it enables so-called Location Based Services (LBS) [2].

In general, user positioning is determined based on Global Navigation Satellite Systems (GNSS), which provide easy to access, globally available positioning services. Using low-cost GNSS receivers is a promising solution for many vehicular applications as they offer a good compromise between reasonable costs for mass production and a good fundamental for many possible approaches to improve positioning accuracy to the point where it is sufficient for applications that require the highest possible accuracies. Due to several error influences, such as satellite orbit, clock biases, atmospheric delays, receiver noise or multipath effects, the possible accuracies of position estimation based exclusively on stand-alone GNSS are in the range of 5 to 10 m in open sky areas [3]. As discussed in Section 2.1.1, accuracy is only one essential performance parameter, as signal availability and positioning integrity are also essential for ADAS and HAD. GNSS based applications regarding performance demands can be classified as:

- **Safety-Critical Applications (SCA)** (also referred to as Safety of Life, SoL) can potentially cause harm to humans, damage the environment or lead to the destruction of the system itself. For road transportation HAD functions or ADAS are prominent examples. Requirements in terms of GNSS performance parameters such as accuracy, availability and integrity are obviously very strict.
- **Liability-Critical Applications (LCA)** were first introduced in Reference [4] and further discussed in Reference [5]. LCA can lead to economic or legal consequences if undetected miss-performances occur. Example applications include GNSS road tolling, fleet management, pay as you drive insurances or law enforcement. These types of applications are technologically enabled by SCA as they provide the necessary GNSS quality of service (QoS) for LCA.
- **Non-Critical Applications (NCA)** are not connected to any kind of health, legal or economic risks for users and their environment. This also leads to less strict performance requirements compared to SCA and LCA. Popular applications are navigational tasks on consumer level.

**Figure 1:**
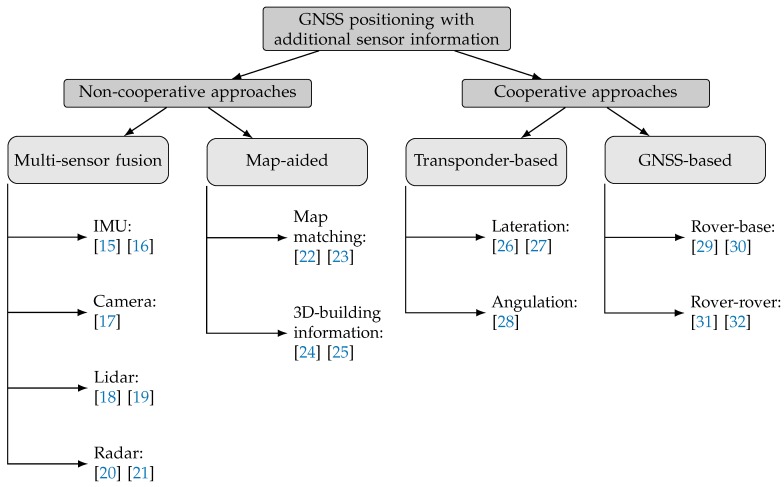
Taxonomy of techniques for improving GNSS performance including related research work.

Necessary performance demand for SCA and LCA according to Reference [6] is an absolute position accuracy (see Section 2.1.2) of 0.7 to 1.1 m at 95% confidence level which is typically not met by stand-alone GNSS positioning techniques. Especially in urban environments, where positioning availability, accuracy and integrity are degraded by non-line-of-sight (NLoS) and multipath occurrence [7], accuracies are further decreased. Overcoming these downsides is usually achieved by incorporating additional sensor respectively environmental information or augmentation systems. A widespread approach for this is the use of on-board sensors. Different types of sensors can provide additional information about the state of the vehicle (e.g., Inertial Measurement Units (IMU), Odometer) and the vehicle environment (e.g., LIDAR, Camera, Radar) [8]. The information gained from multiple sensors can be used by data fusion to improve positioning accuracy [9]. According to Reference [10], all on-board approaches have a Line-of-Sight (LoS) characteristic. Consequently, this means that visual relations can be easily obstructed and thus cause signal loss and deterioration of the results. Cooperative approaches can help to improve this situation. Two different concepts can be distinguished as follows—transponder-based (including wireless sensor measurements) and GNSS-based (building on the exchange of GNSS-related information). A widely used approach to achieve the most accurate absolute position of a rover is to use GNSS-measurements from a base station. In order to make such a method accessible to many road users, a high degree of infrastructure equipment is required. Many road-side units (RSU) acting as base stations would have to be installed on a large scale. In contrast, a relative positioning between road users is being researched. For many SCA in road traffic, no fixed absolute position is assumed at all; rather, knowledge of the distances between each other plays a role. The calculations are primarily based on the exchange of pseudoranges, the so-called pseudorange differencing [11,12]. Other research groups such as those in References [13] or [14] have dealt with the resolution of phase ambiguities based on only one frequency by using carrier phase data. Figure 1 contains a taxonomy of possible techniques and methods for GNSS performance improvements with the aid of additional sensor information at user level as well as current related research work in the mentioned fields [15,16,17,18,19,20,21,22,23,24,25,26,27,28,29,30,31,32]. Accordingly, Reference [8] merges information from GNSS and a camera system with measurements from an IMU, or Reference [33] combines Lidar, IMU and GNSS data.

In this article, a survey on state-of-the-art cooperative positioning (CP) approaches is presented. Past published works of the authors in this research field are References [34,35] where first integration strategies of CP utilizing dedicated short range communication (DSRC) were presented. The here-presented work includes a broader view on this topic, providing additional data, implementational aspects of state-of-the-art positioning methods and a deeper validation of these. In general, the focus of the presented work is solely on positioning performance without the aid of additional sensors or information sources. The results of this contribution can be used as a foundation for more complex positioning and tracking frameworks. Additionally, possible effects that negatively influence positioning performance are discussed and method recommendations for future Vehicle-to-Everything (V2X) enhanced GNSS applications are formulated.

The remainder of the article is structured as follows—Section 2 gives a brief overview on V2X communication, necessary GNSS fundamentals and algorithms, as well as possibilities to enhance GNSS positioning using DSRC, followed by Section 3, which gives a description of the conducted experimental data as well as a comprehensive evaluation of V2X performance and an accuracy assessment of the presented positioning methods. The contribution concludes with a summary and an outlook on possible future research topics.

## 2. Fundamentals

### 2.1. V2X Communication

V2X communication is a generalized term for any form of communication of inter-vehicle (V2V), vehicle-to-infrastructure (V2I), vehicle-to-pedestrians (V2P) or other related devices using radio access technologies. The term C-ITS (Cooperative Intelligent Transport Systems) is used synonymously. For the resulting network based on V2X technology, the term VANET (Vehicular ad-hoc network) was coined. Similar to the already introduced GNSS application classification, V2X communication systems can also be categorized into safety- and non-safety critical applications [36]:

- Safety-critical
  – Emergency vehicle warning and prioritization at intersections
  – Vulnerable road user warning
  – Wrong-way driver detection
  – Cooperative trajectory planing, platooning and collision avoidance
  – Infrastructure and roadwork warning
- Non-safety-critical
  – Traffic light optimal speed advisory
  – Electronic road pricing
  – Infotainment applications
  – Cooperative positioning

**Figure 2:**
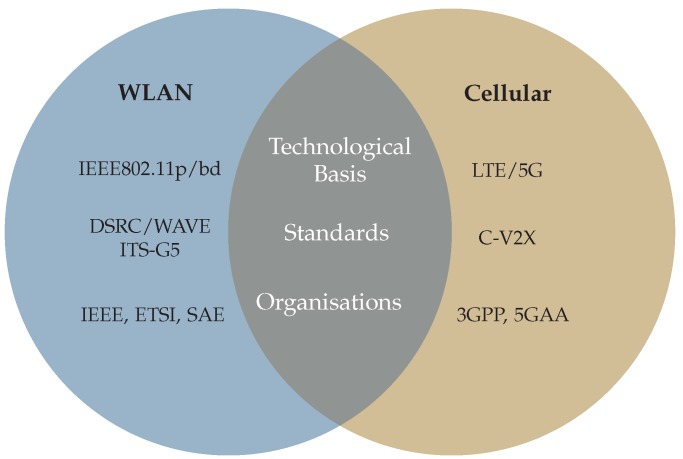
Comparison of wireless local area network (WLAN) and Cellular based Vehicle-to-Everything (V2X) technology.

Radio access technologies (RAT) used for V2X can be categorized in two groups. On the one hand, there are wireless local area network (WLAN) systems based on the IEEE802.11 standard family, most notably IEEE 802.11p. On the other hand, there is the 3rd Generation Partnership Project (3GPP) promoting the Cellular V2X (C-V2X) family, which exploits mobile cellular systems based on 4G/LTE for use in VANETs. Currently, there are attempts to evolve to new generation access technology based on IEEE 802.11bd respectively 5G [37]. An overview is given in Figure 2.

**Figure 3:**
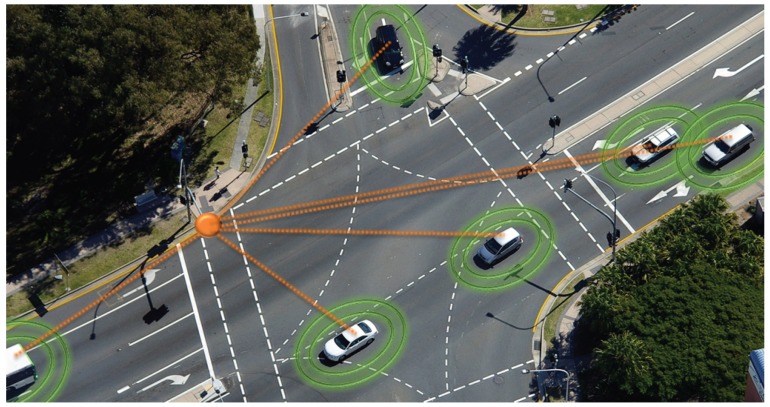
Visualization of a typical VANET cross-road scenario indicating the DSRC broadcast network topology including a stationary road-side unit (orange) and connected vehicles (green).

The standardization landscape on the side of WLAN based technologies is characterized by the two main approaches Dedicated Short Range Communication (DSRC) in the US and C-ITS in Europe. Both exploit the 5.9 GHz frequency band using IEEE 802.11p as RAT. On the European side, the European Telecommunications Standards Institute (ETSI) and European Committee for Standardization (CEN) are the main developers of V2X standards, with ITS-G5 being the central standard family defining messages as well as access technology properties. Main parts of the standard are message definitions. The Cooperative Awareness Message (CAM) is used for continuously disseminating current vehicle states such as position, velocity and status. In contrast, Decentralized Environmental Notification Messages (DENM) are triggered by certain events and are used for communicating safety information in a specified geographic range. Additionally, the Signal Phase & Timing (SPAT) message contain intersection information. DSRC is developed by the DSRC technical committee of the Society of Automotive Engineers (SAE) in cooperation with the IEEE 1609 DSRC working group. In combination with the IEEE 1609 standard family defining networking and application layers, the altogether set is also known as Wireless Access for Vehicular Environment (WAVE). The main message type is the Basic Safety Message (BSM) which serves a similar purpose as the CAM message, including the current vehicle state and movement parameters. Other message types are harmonized with their European counterparts. A typical application of DSRC broadcast in the context of an ITS is displayed in Figure 3.

On the C-ITS side, the ETSI endorsed standard as a basis for V2X applications is the 3GPP release 14, which is considered to be within the 4G/LTE standards range [38]. It incorporates a distributed scheduling mode 4, which allows semi-persistent V2V communication without a centralized network infrastructure. The first 5G NR Release 15 incorporates support for V2X 5G as well and is promoted as the future V2X standard by the 5G automotive alliance. However, this standard and the technological basis can be considered rather experimental, with the disadvantage of limited hardware availability nor extensive testing and application research.

In the current situation, both approaches are concurring for a general acceptance as the V2X standard both legislatively as well as in terms of adaption by the car industry. Concerning the legislation in the European Union, the EU strategy on cooperative, connected and automated mobility proposes a “combination of ETSI ITS-G5 and existing cellular networks”, with “5G playing an important role in the long-term”. However, the current strategy for disseminating V2X technology in Europe has not been decided on a technological basis yet [39]. As ITS-G5 is considered to be more ready-to-use compared to C-V2X standards [40], we decided to use this technological basis for further investigation, albeit the appliance of C-V2X technology would be equally possible within the same cooperative positioning approach.

Evaluating V2X communication performance is often achieved by examining the Received Signal Strength (RSS) of the received packets [41,42]. The RSS is a sufficient starting point for radio channel modelling as it provides a rough estimate of reception conditions. In recent years, References [43,44] and [45] have also investigated the radio propagation channel in vehicular environments. A theoretical basis for RSS $P_{\mathrm{Rx}}$ is provided by the link budget which balances the signal power on receiver site, determining and evaluating the transmission quality. It is simplified defined as [46]:

(1) $$P_{\mathrm{Rx}}=P_{\mathrm{Tx}}+G_{\mathrm{Tx}}-L_{\mathrm{Tx}}-L_{\mathrm{FSPL}}+G_{\mathrm{Rx}}-L_{\mathrm{Rx}},$$

where $P_{\mathrm{Tx}}$ represents the transmitter’s transmission power, $G_{\mathrm{Tx}}$ denotes the transmitter’s antenna gain and $L_{\mathrm{Tx}}$ the transmitter losses caused, for example, by cable attenuation or connectors. The combination of $P_{\mathrm{Tx}}$, $G_{\mathrm{Tx}}$ and $L_{\mathrm{Tx}}$ is also referred to as Equivalent Isotropically Radiated Power (EIRP). Similarly, $L_{\mathrm{Rx}}$ represent the connection loss and $G_{\mathrm{Rx}}$ the antenna gain on receiver side. Finally, the free space path loss (FSPL) describes a model for attenuation of RF signals assuming an obstacle free transmission between transmitter and receiver. The existing path loss $L_{\mathrm{FSPL}}\left[\mathrm{dB}\right]$ is then defined as:

(2) $$L_{\mathrm{FSPL}}=\frac{P_{\mathrm{Rx}}}{P_{\mathrm{Tx}}}=20\log_{10}\left(\frac{4\pi d}{\lambda }\right)$$

with *d* denoting the distance between transmitter and receiver and λ being the signal wavelength depending on the signal frequency.

#### 2.1.1. GNSS Fundamentals

The core principle of GNSS positioning is to synchronously measure distances between a GNSS receiver, whose position has to be determined, and multiple satellites with known positions using radio-frequency (RF) signals. These measurements are either based on measuring signal run-times between the receiver and the satellites, also referred to as code-phase measurements, or determining the number of phase cycles given a known signal wavelength. The second measurement principle is also referred to as carrier-phase measurement. As already mentioned in Section 1, the presented work focuses on low-cost single frequency code-phase measurements, which are also referred to as pseudoranges (PR) ρ. To compute a receiver position based on PR estimations the following state vector $x_{r}$ has to be resolved:

(3) $$x_{r}={\left[X\;Y\;Z\;\delta t_{r}\right]}^{⊺}$$

consisting of the rovers three-dimensional position coordinates as well as the receiver clock offset. Determining these unknowns using PR requires at least four receivable GNSS signals. The underlying functional model describes PR measurements between satellites *s* and receiver *r* [3]:

(4) $$\rho_{r}^{s}=d_{r}^{s}-\left(\delta t_{r}-\delta t^{s}\right)c+\delta_{\mathrm{ion}}+\delta_{\mathrm{trop}}+\varepsilon$$

where *c* represents the speed of light, $\delta t^{s}$ and $\delta t_{r}$ the receiver’s and the satellite’s clock offset, $\delta_{\mathrm{ion}}$ and $\delta_{\mathrm{trop}}$ the ionospheric and tropospheric error component. All unmodeled error, more specifically uncorrelated errors—for example, receiver noise or multipath influence—are contained in ε. Finally, $d_{r}^{s}={∥x^{s}-x_{r}∥}_{2}$ is the true spatial distance between *r* and *s*, which are given by the their respective position vectors $x^{s}={\left[X^{s},Y^{s},Z^{s}\right]}^{⊺}$ and $x_{r}={\left[X,Y,Z\right]}^{⊺}$ in a three-dimensional Cartesian earth centered earth fixed (ECEF) coordinate system. Common methods for estimating the $x_{r}$ are discussed in Section 2.1.1.

The most common techniques for estimating user position and clock bias are realizations of the Least Squares Estimator (LSE) and the (Extended) Kalman Filter (KF; EKF). In general, the LSE estimates each given observation epoch independently of each other, whereas the EKF is a sequential estimation method which allows the use of process knowledge as well as the information fusion with, for example, vehicle dynamic data. In contrast, both the LSE and KF require linear models as a necessary prerequisite for method applicability. As the GNSS state vector and the underlying measurements (Equations (3) and (4)) are related non-linearly, this is overcome by linearizing Equation (4) using Taylor’s approximation. The operating point for linearization is given by the last available position estimation ${\hat{x}}_{r}$. This leads to the measurement matrix $J_{H}$ as the Jacobian of user position and clock bias as given in Equation (5). This Jacobian matrix is also referred to as the design matrix A. Another common denotation for A is G, referring to it as the geometry matrix.

(5) $$\nabla H=J_{H}={\left(\begin{array}{cccc} \frac{\partial \rho_{1}}{\delta X} & \frac{\partial \rho_{1}}{\delta Y} & \frac{\partial \rho_{1}}{\delta Z} & \frac{\partial \rho_{1}}{\delta \partial_{t_{r}}}\\ \frac{\partial \rho_{2}}{\delta X} & \frac{\partial \rho_{2}}{\delta Y} & \frac{\partial \rho_{2}}{\delta Z} & \frac{\partial \rho_{2}}{\delta \partial_{t_{r}}}\\ \vdots & \vdots & \vdots & \vdots \\ \frac{\partial \rho_{n}}{\delta X} & \frac{\partial \rho_{n}}{\delta Y} & \frac{\partial \rho_{n}}{\delta Z} & \frac{\partial \rho_{n}}{\delta \partial_{t_{r}}} \end{array}\right)}_{n\times 4}={\left(\begin{array}{c} {\left(e_{\mathrm{est}}^{S1}\right)}^{⊺}\;1\\ {\left(e_{\mathrm{est}}^{S2}\right)}^{⊺}\;1\\ \vdots \\ {\left(e_{\mathrm{est}}^{n}\right)}^{⊺}\;1 \end{array}\right)}_{n\times 4}=A.$$

Following the LSE approach, the directional vector between *r* and *s* is normalized as unit directional vector using:

(6) $$e_{\mathrm{est}}^{s}=\frac{{\hat{x}}_{r}-x^{s}}{{\left|\left|{\hat{x}}_{r}-x^{s}\right|\right|}_{2}}.$$

For position estimation using LSE, this leads to the measurement equation for the number of satellites *n*:

(7) $$\delta \rho_{\mathrm{corr}}=A_{n\times 4}{\left(\begin{array}{c} \delta x_{r}\\ \delta t_{r} \end{array}\right)}_{4\times 1}+\epsilon_{n\times 1}.$$

According to the LSE, minimizing the PR residuals $\epsilon_{s}$ are given by:

(8) $$\epsilon_{s}={\left|\left|\delta \rho_{\mathrm{corr}}-A\left(\begin{array}{c} \delta {\hat{x}}_{r}\\ \delta {\hat{t}}_{r} \end{array}\right)\right|\right|}_{2}.$$

Additionally, a stochastic covariance weighting matrix W, which leads to the method terminology Weighted Least Squares Estimator (WLSE), can be applied. As it will be discussed in Section 3, a-priori known NLOS satellites can be weighted using this approach. Furthermore, the weighting can be performed based on the satellites elevation or carrier to noise ratio (CNR). The resulting innovation vector y is calculated as follows:

(9) $$\hat{y}=\left(\begin{array}{c} \delta {\hat{x}}_{r}\\ \delta {\hat{t}}_{r} \end{array}\right)={\left(A^{⊺}WA\right)}^{-1}A^{⊺}W\delta \rho_{\mathrm{corr}}.$$

Applying $\hat{y}$, the system states at time step *k* can be formulated based on the last estimate $x_{r_{k-1}}$ as:

(10) $$x_{r_{k}}=x_{r_{k-1}}+\delta {\hat{x}}_{r}.$$

This approach is referred to as Single Positioning LSE (SP-LSE).

In contrast to the just-introduced LSE based single epoch state estimation, the KF, as introduced in Reference [47], is a Recursive Bayes Filter (RBF) providing a sequential estimation method determining the parameters of a Gaussian probability density function given a systems state vector x at a certain time step *k* following a prediction and correction structure.

As pointed out in Reference [48], the KF provides a suitable and easy to implement solution for a variety of state estimation problem statements. Originally, the applicability of the KF assumes that the underlying process information and given measurements can be formulated with linear equations:

(11) $$x_{k}=\Phi_{k-1}x_{k-1}+w_{k-1}$$

(12) $$z_{k}=H_{k}x_{k}+v_{k}.$$

In Equation (11), Φ represents the state transition matrix, containing the physical relations between state variables. H in Equation (12) denotes the measurement matrix which describes the relations between state variables and available measurements. Additionally to a linear system description, process and measurement noise, $w_{k}$ and $v_{k}$, is assumed to be zero mean multivariate Gaussian white noise with given process noise covariance $\Sigma_{Q}$ and measurement noise covariance $\Sigma_{R}$:

(13) $$\begin{array}{ccc} w_{k}∼N\left(0,\Sigma_{Q_{k}}\right) & \; & v_{k}∼N\left(0,\Sigma_{W_{k}}\right). \end{array}$$

In addition to recursive estimation of the state variables, these covariance matrices are used to estimate the filter’s (co-)variances $\Sigma_{P}$.

Since many state estimation applications are nonlinear, a linearization has to be performed. This can affect both the state transition and the measurement matrix. Aforementioned linearization is achieved by performing a Taylor approximation, similar to the aforementioned LSE approach. A precondition for applicability is that Φ and H are differentiable.

The applications presented in this contribution describe linear process models, whereas estimating user position based on PR is obviously nonlinear. Therefore, the basics of an EKF are briefly described. First, the linearization of $H_{k}$ considering the last estimation of the state vector $\hat{x}$ is performed according to Equation (5). After an initialization of the state vector and corresponding covariance matrices, the EKF recursively follows a prediction and correction structure. Initialization can be performed with respect to a-priori available knowledge. Filter design and parameterization will be discussed in Section 2.3 according to the specific application cases. An overview of all necessary components, equations and computation steps is given in Table 1 where *n* represents the number of state variables and *m* the number of available PR measurements.

Both introduced position estimation approaches will be utilized for CP approaches which will be introduced in Section 2.2.

#### 2.1.2. GNSS Accuracy Assessment

The performance of GNSS positioning is generally measured by the following parameters—accuracy, integrity, continuity and availability. These parameters are not independent of each other but rather build hierarchically upon each other. The basis is the availability of sufficient GNSS signals. A steady positioning is only possible by the continuity of the received signals. Availability and continuity are therefore the long- and short-term considerations of the same parameter. Additionally, even if continuity is guaranteed, the system can only be used for safety-critical applications if the observables are not systematically wrong, that is, the integrity of the signals is guaranteed. Based on this, the accuracy can be defined as the strictest criterion which is the sole focus of this contribution. Further discussion will be conducted in Section 2.1.2.

For validation and evaluation of GNSS accuracy performance, statistical metrics and measures are utilized. Generally, this assessments can be performed in three different domains:

- Position domain
- Measurement domain
- Satellite constellation domain

In the position domain, let $x_{\mathrm{true}}$ denote the real two- or three-dimensional user position and $\hat{x}$ denote the estimated user position. The positioning error is then defined as the offset between $x_{\mathrm{true}}$ and $\hat{x}$. Suitable quality measures for expressing position accuracy are the Mean Absolute Error (MAE) and the Root Mean Square Error (RMSE) [49]. Single epoch absolute error and root square error will further on be referred to as AE and RSE. For *N* of measured epochs and with k=1,2,…,N, these are defined as:

(14) $$Q_{\mathrm{MAE}}=\frac{1}{N}\sum_{k}{\left|\left|x_{{\mathrm{true}}_{k}}-{\hat{x}}_{k}\right|\right|}_{1}$$

(15) $$Q_{\mathrm{RMSE}}=\sqrt{\frac{1}{N}\sum_{k}{\left|\left|x_{{\mathrm{true}}_{k}}-{\hat{x}}_{k}\right|\right|}_{2}}$$

Additionally, errors in the measurement domain, as indicated by Equation (4), are negatively influencing PR measurements. These include clock biases as well as atmospheric and stochastic errors. Additionally, satellite ephemeris and orbit faults can occur. Combining these aforementioned error components forms the GNSS error budget associated with a certain confidence. The error budget is expressed using the User Equivalent Range Error (UERE, $Q_{\mathrm{UERE}}$) which is defined as a systematic accuracy quantity for PR measurements [3]. A quantitative example of UERE for GPS Standard Positioning Service (SIS) is given in Reference [50] is given by $Q_{\mathrm{UERE}}=7.8\;m$.

Furthermore, an evaluation of the underlying satellite constellation can be conducted using the concept of Dilution of Precision (DOP). The DOP values, Geometric-, Position-, Horizontal-, Vertical- and Time-DOP (GDOP, PDOP, HDOP, VDOP and TDOP), provide measures for the influence of satellite geometry on the positioning result. The basis for DOP calculation is the covariance matrix of $\hat{y}$ from Equation (9) containing variances $\sigma^{2}$ and covariances of the state vectors components (see Equation (3)), which can be calculated using the geometry matrix $\mathrm{cov}\left(\hat{y}\right)={\left(A^{⊺}A\right)}^{-1}$ [3]. Utilizing $\mathrm{cov}(\hat{y})$, DOP calculation can be performed as follows:

(16) $$\begin{array}{ccc} \begin{array}{c} \mathrm{GDOP}=\sqrt{trace(\mathrm{cov}\left(\hat{y}\right))}\\ \mathrm{PDOP}=\sqrt{\sigma_{X}^{2}+\sigma_{Y}^{2}+\sigma_{Z}^{2}} \end{array}\; & \; & \begin{array}{c} \mathrm{HDOP}=\sqrt{\sigma_{e}^{2}+\sigma_{n}^{2}}\\ \mathrm{VDOP}=\sqrt{\sigma_{u}^{2}} \end{array} \end{array}$$

HDOP and VDOP calculation is typically performed in the user plane. A coordinate transformation to a local Earth-North-Up (ENU) frame is necessary using the user’s geographical coordinates latitude and longitude and rotation matrix [3].

### 2.2. Cooperative Positioning

**Figure 4:**
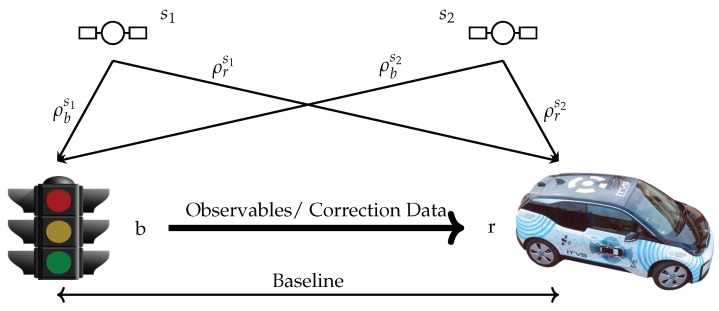
Basic VANET cooperative positioning approach.

CP describes several approaches which combine and fuse different position related information based on a communication link. Reference [51] distinguishes between conventional and modern CP methods. Conventional CP includes classical differential GNSS (DGNSS) approaches, which were first introduced in the 1980s. On the other hand, modern CP approaches incorporate vehicular communication. For the presented contribution, CP is limited to the exchange of GNSS observation data between several ITS-stations used in a VANET as exemplary shown in Figure 4.

The combination of multiple observations from different receivers and satellites is referred to as differencing. These approaches allow the elimination of correlated error influences including ionospheric, tropospheric and satellite clock/orbit errors without the need of quantification. Differencing techniques are generally distinguished between the number of constituted differences. The following sections will present single (SD) and double differences (DD) which are typically applicable for modern CP methods, like in References [51,52].

Similar to Section 2.1.2, differencing can be performed in both the position and the measurement domain. Other than that, differences can be performed between different receiver and satellite constellations while also incorporating diverse types of measurements. For PR based approaches, these include:

- Between receiver differencing (BRD)
  – Fixed base station
  – Moving base station
  – Fixed baseline
- Between satellite differencing (BSD)

This contribution focuses on positioning following the BRD approach with a fixed position reference station. The underlying configuration is referred to as a rover base constellation. A fixed base station, which is used as a reference station with a known position, is utilized. The rover is the vehicle whose position needs to be estimated. A typical application scenario is an RSU, which is equipped with both a GNSS receiver and a DSRC module to broadcast its observables respective to correction data to surrounding connected vehicles.

#### 2.2.1. Single Differencing

SD describes methods which use observation from a set of satellites, the same as at least two GNSS receivers (BRD) or form differences between multiple satellite observations to one receiver (BSD). When using only PR measurements, the former SD is commonly referred to as DGNSS. Again, this differencing can be performed in both the position and measurement domains.

For SD methods in the position domain, both the base ${\hat{p}}_{b}$ and rover ${\hat{p}}_{r}$ positions are estimated independently. At the fixed base station, these estimations are compared with the a-priori known reference position $p_{b}$ of the base station. The positional offset is given by $\Delta p_{b}=p_{b}-\hat{p_{b}}$. The determined positional correction can then be applied to the estimated rover position $\hat{p_{r}}=\hat{p_{r}}+\Delta p_{b}$. This method is also referred to as absolute position differencing (APD).

The second approach, PR differential, aims to correct single PR measurements which are received at both rover and base stations. BRD SD, also called between receiver single differencing (BRSD), can mathematically be expressed as follows:

(17) $$SD_{r,b}^{s}=\rho_{r}^{s}-\rho_{b}^{s}.$$

Applying Equation (4) to Equation (17) leads to:

(18) $$SD_{r,b}^{s}=d_{r}^{s}-d_{b}^{s}-c\left(\delta t_{r}-\delta t_{b}\right)+c\left(\delta t^{s}-\delta t^{s}\right)+\underset{=0\;\mathrm{for}\;\mathrm{short}\;\mathrm{baseline}}{\underset{︸}{\Delta \delta_{\mathrm{ion}}+\Delta \delta_{\mathrm{trop}}}}+\;\mathrm{cov}\left(\varepsilon_{r},\varepsilon_{rb}\right)$$

(19) $$SD_{r,b}^{s}=d_{r}^{s}-d_{b}^{s}-c\left(\delta t_{r}-\delta t_{b}\right)+\mathrm{cov}\left(\varepsilon_{r},\varepsilon_{b}\right).$$

For practical implementation, the first computational step at base side is to determine the offset of each PR measurement to the known true spatial distance between the reference station and each satellite. This offset can be interpreted as a PR correction, which is then transmitted and applied at rover side. As shown in Equations (18) and (19) satellite clock biases get eliminated for the same satellite set in view. Assuming a short baseline between master and rover, atmospheric error influences are correlated and therefore their difference is insignificantly small. The remaining error terms are the difference in base and rover clock bias as well as the stochastic influences on both stations.

#### 2.2.2. Double Differencing

To further eliminate the receiver clock bias, DD can be applied. Similar to SD, DD is the combination of observations between two GNSS receivers. Additionally, differences between satellites $s_{1}$ and $s_{2}$ are also calculated. In addition to Equation (17) this leads to:

(20) $$DD_{r,b}^{s_{1},s_{2}}=\left(\rho_{r}^{s_{1}}-\rho_{b}^{s_{1}}\right)-\left(\rho_{r}^{s_{2}}-\rho_{b}^{s_{2}}\right)$$

(21) $$\begin{array}{c} DD_{r,b}^{s_{1},s_{2}}=dr^{s_{1}}-d_{r}^{s_{2}}-d_{b}^{s_{1}}+d_{b}^{s_{2}}+c(\delta t_{r}-\delta t_{b})-c(\delta t_{r}-\delta t_{b})+c\left(\delta t^{s_{1}}-\delta t^{s_{1}}\right)\\ -c\left(\delta t^{s_{2}}-\delta t^{s_{2}}\right)+\underset{=0\;\mathrm{for}\;\mathrm{short}\;\mathrm{baseline}}{\underset{︸}{\Delta \delta_{\mathrm{ion}}+\Delta \delta_{\mathrm{trop}}}}+\;\mathrm{cov}\left(\varepsilon_{r},\varepsilon_{b}\right) \end{array}$$

(22) $$\begin{array}{c} DD_{r,b}^{s_{1},s_{2}}=d_{r}^{s_{1}}-d_{r}^{s_{2}}-d_{b}^{s_{1}}+d_{b}^{s_{2}}+\;\mathrm{cov}\left(\varepsilon_{r},\varepsilon_{b}\right). \end{array}$$

As indicated by Equations (21) and (22), the corresponding receiver clock offsets $\delta t_{r}$ and $\delta t_{b}$ are eliminated as well. As the unmodelled error, which is modelled as Gaussian white noise, is further increased due to covariance propagation compared to SD, DD can generally be considered to yield no performance gain. If this error component is increased by, for example, multipath effects, DD may even perform significantly worse than SD and SP. However, DD enables solving mechanisms for carrier-phase integer ambiguities, making it a prerequisite for the highly accurate real-time kinematic (RTK) positioning technique.

#### 2.2.3. CP Summary

Observation combination represents a state-of-the-art approach to reduce or eliminate correlated error terms on base and rover station PR measurements, given a short baseline. Integrating this approach utilizing a VANET infrastructure also supplies several benefits. VANETs provide a locally available communication link, which realizes the criteria for ultra short baselines for observation differencing. Additionally, VANET DSRC is a broadcast link, conveniently allocating available information to all agents in reach.

While eliminating correlated error terms with observation combination, the negative influence of unmodelled stochastic errors increases with each differencing step applied [53,54]. Especially in urban environments where NLoS reception and multipath scenarios are likely to occur, this error decorrelation between rover and base station can lead to significant position outliers up to 100 m [55]. This is caused by covariance error propagation induced by each differencing step, which increases the influence of ε [53] (see Table 2) since the influence of ε increases [53]. Additionally, data differences are only applicable to the same satellite set in view, potentially eliminating the number of satellites available at each respective station. Table 2 gives a detailed overview of differencing steps as well as their consequences on different error terms. A detailed discussion of low-cost based CP differencing and its effects on positioning accuracy is given in Section 3.

### 2.3. Implementation Aspects & Filter Design

For the implementation of BSD approaches (including DD), a reference satellite (indicated by superscript *M*) was selected. Usually, the satellite with the highest elevation is predestined to be used as reference satellite since it is assumed to provide the best geometric constellation and the least amount stochastic error influence [56]. This approach is also followed in the realization of BSD methods in this contribution. As an example, continuative approaches on reference satellite selection to lower induced noise can be found in Reference [57].

In the following, the EKF filter design will be discussed. The state vectors for GNSS position estimation depending on the positioning mode are defined as:

(23) $$\begin{array}{c} \begin{array}{c} x^{\mathrm{SP}}={\left[X\;\dot{X}\;Y\;\dot{Y}\;Z\;\dot{Z}\;\delta t_{r}\;\delta \dot{t_{r}}\right]}^{⊺} \end{array} \end{array}$$

(24) $$\begin{array}{c} \begin{array}{c} x^{\mathrm{SD}}={\left[X\;\dot{X}\;Y\;\dot{Y}\;Z\;\dot{Z}\;\delta t_{b-r}\;\delta {\dot{t}}_{b-r}\right]}^{⊺} \end{array} \end{array}$$

(25) $$\begin{array}{c} \begin{array}{c} x^{\mathrm{DD}}={\left[X\;\dot{X}\;Y\;\dot{Y}\;Z\;\dot{Z}\right]}^{⊺} \end{array} \end{array}$$

While all EKF variations estimate the three-dimensional position components and their respective derivatives (velocities), the state vectors differ in receiver clock drift modelling. For SP applications, the receiver clock bias is directly modelled and estimated. Since the bias is time varying, an estimation of its drift is performed [58]. In contrast, applying single differences between base station and rover leads to an estimation of the bias difference between both. Since all receiver clock errors are eliminated applying DD, these components are no longer included in the respective state vector.

At first, the EKF prediction step is introduced. For further implementation, all EKF are based on a Constant Velocity (CV) motion model. The corresponding state transition matrices for SP and SD are given as:

(26) $$\Phi^{\mathrm{SP};\mathrm{SD}}=\left(\begin{array}{cccc} F_{\mathrm{block}} & 0_{2\times 2} & 0_{2\times 2} & 0_{2\times 2}\\ 0_{2\times 2} & F_{\mathrm{block}} & 0_{2\times 2} & 0_{2\times 2}\\ 0_{2\times 2} & 0_{2\times 2} & F_{\mathrm{block}} & 0_{2\times 2}\\ 0_{2\times 2} & 0_{2\times 2} & 0_{2\times 2} & F_{\mathrm{block}} \end{array}\right)\;\mathrm{where}\;F_{\mathrm{block}}=\left(\begin{array}{cc} 1 & \Delta t\\ 0 & 1 \end{array}\right),$$

with $0_{2\times 2}$ representing a square zero matrix of dimension 2 and Δt being the time interval between measurements. For DD applications, all receiver clock biases are eliminated (see Section 2.2). Therefore $\Phi^{\mathrm{DD}}$ is structurally similar to $\Phi^{\mathrm{SP}}$ and $\Phi^{\mathrm{SD}}$, but different in $rank(\Phi^{\mathrm{DD}})=6$.

The corresponding matrices Q for SP, SD and DD is given by:

(27) $$\begin{array}{ccc} \Sigma_{Q^{\mathrm{SP}}}=\Sigma_{Q^{\mathrm{SD}}}=\left(\begin{array}{cccc} \Sigma_{Q^{\mathrm{pv}}} & 0_{2\times 2} & 0_{2\times 2} & 0_{2\times 2}\\ 0_{2\times 2} & \Sigma_{Q^{\mathrm{pv}}} & 0_{2\times 2} & 0_{2\times 2}\\ 0_{2\times 2} & 0_{2\times 2} & \Sigma_{Q^{\mathrm{pv}}} & 0_{2\times 2}\\ 0_{2\times 2} & 0_{2\times 2} & 0_{2\times 2} & \Sigma_{Q^{\mathrm{Clk}}} \end{array}\right)\; & \; & \Sigma_{Q^{\mathrm{SP}}}=\left(\begin{array}{ccc} \Sigma_{Q^{\mathrm{pv}}} & 0_{2\times 2} & 0_{2\times 2}\\ 0_{2\times 2} & \Sigma_{Q^{\mathrm{pv}}} & 0_{2\times 2}\\ 0_{2\times 2} & 0_{2\times 2} & \Sigma_{Q^{\mathrm{pv}}} \end{array}\right) \end{array}$$

where

(28) $$\begin{array}{ccc} \Sigma_{Q^{\mathrm{pv}}}=\left(\begin{array}{cc} \frac{S_{p}\Delta t^{3}}{2} & \frac{S_{p}\Delta t^{2}}{2}\\ \frac{S_{p}\Delta t^{2}}{2} & S_{p}\Delta t \end{array}\right)\; & \; & \Sigma_{Q^{\mathrm{Clk}}}=\left(\begin{array}{cc} S_{f}\Delta t+\frac{S_{g}\Delta t^{3}}{3} & \frac{S_{g}\Delta t^{2}}{2}\\ \frac{S_{g}\Delta t^{2}}{2} & S_{g}\Delta t. \end{array}\right) \end{array}$$

In the process covariance matrices $\Sigma_{Q}$, the position-velocity blocks are described as a random process characterized by the spectral amplitude $S_{p}$ based on the expected vehicle dynamics. Discussion of proper parametrizations are given in References [59]. As far as receiver clock bias and drift modelling is concerned, a two-state random process is assumed as described in Reference [58]. Parametrizations for the amplitudes $S_{f}$ and $S_{g}$ are also given there in dependence on the assumed clock quality.

In line with the process models, SP and SD follow a similar measurement model. First of all, the Jacobian matrix is defined as:

(29) $$J_{H}^{SP;SD}=\left(\begin{array}{cccccccc} \frac{\partial \rho^{1}}{\delta X} & 0 & \frac{\partial \rho^{1}}{\delta Y} & 0 & \frac{\partial \rho^{1}}{\delta Z} & 0 & 1 & 0\\ \frac{\partial \rho^{2}}{\delta X} & 0 & \frac{\partial \rho_{2}}{\delta Y} & 0 & \frac{\partial \rho^{2}}{\delta Z} & 0 & 1 & 0\\ \vdots & \vdots & \vdots & \vdots & \vdots & \vdots & \vdots & \vdots \\ \frac{\partial \rho^{n}}{\delta X} & 0 & \frac{\partial \rho^{n}}{\delta Y} & 0 & \frac{\partial \rho^{n}}{\delta Z} & 0 & 1 & 0. \end{array}\right)$$

For BSD approaches, differencing also affects the design matrix which for all satellites *s* except the reference satellite M∉s is given by:

(30) $$J_{H}^{DD}=\left(\begin{array}{cccccc} \frac{\partial \rho^{1}}{\delta X}-\frac{\partial \rho^{M}}{\delta X} & 0 & \frac{\partial \rho^{1}}{\delta Y}-\frac{\partial \rho^{M}}{\delta Y} & 0 & \frac{\partial \rho^{1}}{\delta Z}-\frac{\partial \rho^{M}}{\delta Z} & 0\\ \frac{\partial \rho^{2}}{\delta X}-\frac{\partial \rho^{M}}{\delta X} & 0 & \frac{\partial \rho_{2}}{\delta Y}-\frac{\partial \rho^{M}}{\delta Y} & 0 & \frac{\partial \rho^{2}}{\delta Z}-\frac{\partial \rho^{M}}{\delta Z} & 0\\ \vdots & \vdots & \vdots & \vdots & \vdots & \vdots \\ \frac{\partial \rho^{n}}{\delta X}-\frac{\partial \rho^{M}}{\delta X} & 0 & \frac{\partial \rho^{n}}{\delta Y}-\frac{\partial \rho^{M}}{\delta Y} & 0 & \frac{\partial \rho^{n}}{\delta Z}-\frac{\partial \rho^{M}}{\delta Z} & 0. \end{array}\right)$$

The respective innovation vectors $y^{\mathrm{SP}}$, $y^{\mathrm{SD}}$ and $y^{\mathrm{DD}}$ are defined as (M∉s applies to $y^{\mathrm{DD}}$):

(31) $$y^{\mathrm{SP}}=\left(\begin{array}{c} \rho_{r}^{1}-{∥x^{1}-x_{r}∥}_{2}+\delta_{\mathrm{ion}}^{1}+\delta_{\mathrm{trop}}^{1}-c\;\cdot \;\delta t^{1}\\ \vdots \\ \rho_{r}^{n}-{∥x^{n}-x_{r}∥}_{2}+\delta_{\mathrm{ion}}^{n}+\delta_{\mathrm{trop}}^{n}-c\;\cdot \;\delta t^{n} \end{array}\right)$$

(32) $$y^{\mathrm{SD}}=\left(\begin{array}{c} \rho_{r}^{1}-{∥x^{1}-x_{r}∥}_{2}-\rho_{b}^{1}+{∥x^{1}-x_{b}∥}_{2}\\ \vdots \\ \rho_{r}^{n}-{∥x^{n}-x_{r}∥}_{2}-\rho_{b}^{n}+{∥x^{n}-x_{b}∥}_{2} \end{array}\right)$$

(33) $$y^{\mathrm{DD}}=\left(\begin{array}{c} \rho_{r}^{1}-\rho_{r}^{M}-\rho_{b}^{1}+\rho_{b}^{M}-{∥x^{1}-x_{r}∥}_{2}+{∥x^{1}-x_{b}∥}_{2}+{∥x^{M}-x_{r}∥}_{2}-{∥x^{M}-x_{b}∥}_{2}\\ \vdots \\ \rho_{r}^{n}-\rho_{r}^{M}-\rho_{b}^{n}+\rho_{b}^{M}-{∥x^{n}-x_{r}∥}_{2}+{∥x^{n}-x_{b}∥}_{2}+{∥x^{M}-x_{r}∥}_{2}-{∥x^{M}-x_{b}∥}_{2}. \end{array}\right)$$

Lastly, $\Sigma_{R}$ can be defined as:

(34) $$\Sigma_{R}=\omega \;\cdot \;Q_{\mathrm{UERE}}\;\cdot \;I_{n\times n},$$

with ω=1,2,4 (see Table 2) for $\Sigma_{R}^{\mathrm{SP}}$, $\Sigma_{R}^{\mathrm{SD}}$ and $\Sigma_{R}^{\mathrm{DD}}$ respectively, indicating variance propagation.

## 3. Results & Discussion

### 3.1. Data Foundation

**Figure 5:**
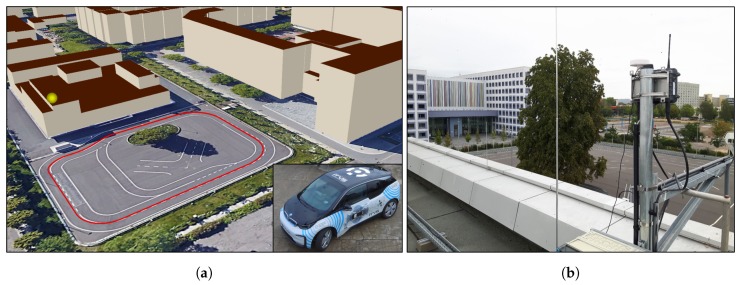
Illustration of the reference trajectory and its surroundings: (**a**) Testbed for automated and connected driving including 3D building information, base station position (yellow), reference track (red) and test vehicle (right, ©GoogleEarth); (**b**) Roof-mounted GNSS base station and DSRC RSU (point-of-view from base station towards the building displayed on the right side of (**a**)).

The measurements presented in this contribution were conducted on a closed testbed for connected and autonomous driving located in Dresden, Germany. An illustration of the area and its urban surrounding is displayed in Figure 5. For the test drive, the targeted trajectory was the testbed’s center line indicated by the red line. In total, a drive of about one hour was performed, collecting 6199 epochs given a measurement rate of 2Hz. All provided data were evaluated in a post-processing manner.

**Figure 6:**
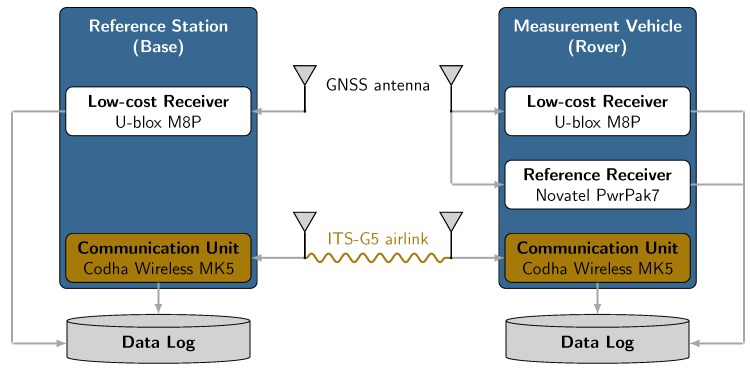
Schematic measurement setup.

As indicated by Figure 6, the base station consists of a *u-blox M8P* single frequency GNSS receiver equipped with its accompanying GNSS antenna connected via USB to a notebook for data logging. Additionally, a *Cohda Wireless MK5* RSU was used to transmit GNSS observation respectively correction data. On rover side, the test vehicle was equipped with a *Cohda Wireless MK5* OBU, logging all received packets as well as their Received Signal Strength Indicator (RSSI). Just like for the base station, a *u-blox M8P* GNSS receiver was used. At both stations, message types *UBX-RXM-RAWX* for PR measurements and *UBX-RXM-SFRBX* for ephemeris data for GPS and GLONASS satellites were recorded. Obtaining a ground truth serving as a reference trajectory was achieved by using a *NovAtel PwrPak7* receiver. The receiver’s *BestPos* message with position type *INS SBAS*, indicating a SBAS aided INS-GNSS sensor data fusion position solution, was logged.

**Figure 7:**
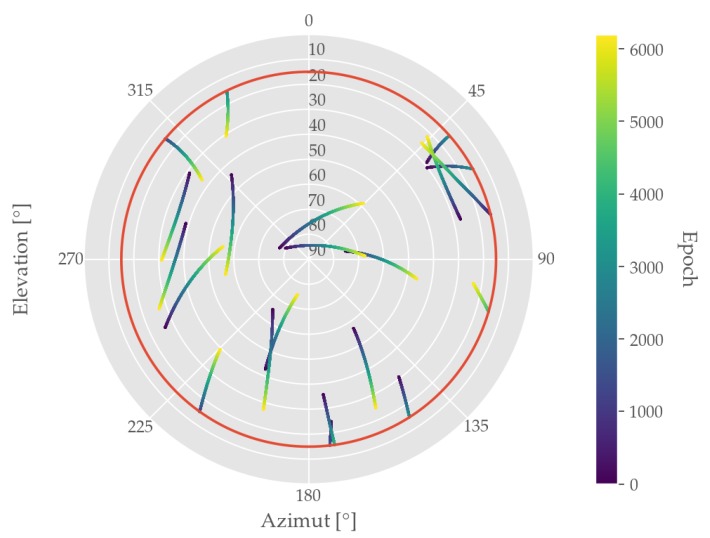
Skyplot of collected GNSS dataset showing the applied elevation mask (red) and the Azimuth and Elevation angles of all observed satellites given the respective epoch.

As far as available GNSS measurements are concerned, both GPS and GLONASS satellites were observed. During the entire test run an average number of satellites $\bar{n}=16$ was received, which will be discussed later on. A further assessment of the underlying satellite constellation is given in Figure 7, which displays the skyplot of the observed GNSS data as well as the applied elevation mask of $\theta =15{}^{{}^{\circ}}$. As the satellites are scattered over the visible sky, this leads to a good geometrical resolution resulting in comparably low DOP values with an average HDOP of 1.1, which can be found in Table 3.

### 3.2. V2X Communication

**Figure 8:**
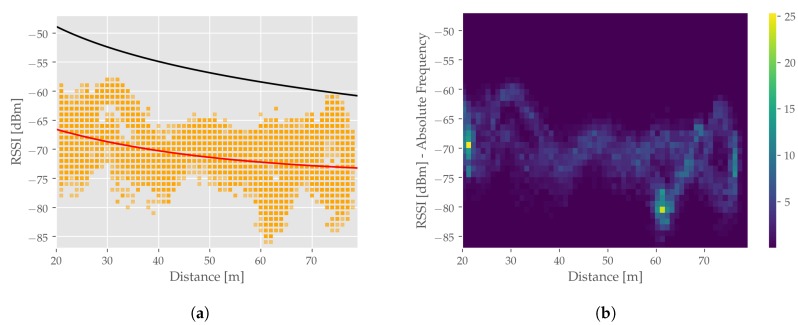
Visualization of RSSI measurements collected during the measurement run—(**a**) Theoretical RSS applying FSPL (black), Measured RSSI (orange), Derived RSS approximation based on measured RSSI (red). (**b**) Absolute frequency of RSSI values indicated by the colormap brightness.

In order to evaluate the VANET performance, the underlying physical parameters as well as the performance with regard to the given application are evaluated. During the entire test run no *rawITS* package between base and rover was dropped, resulting in a 100% Packet Success Rate (PSR). To further validate the communication performance given the utilized setup, the Received Signal Strength (RSSI) between base and rover is examined. The RSSI is a vendor-specific measure and provides an internally processed indication of the RSS. RSS and RSSI will further on be regarded as equivalent as a performed calibration measurement measuring the reception power using a spectrum analyzer and the RSSI using the radio modules revealed no substantial deviations. A depiction of measured RSSI in the measurement scenario is given in Figure 8.

Figure 8a shows the difference between theoretical RSS modelling the path loss applying a FSPL and the measured RSSI. For the calculation of the maximal reception power, the following transmission power, antenna gains and connection losses were considered: $P_{\mathrm{Tx}}=23\;\mathrm{dBm}$, $G_{\mathrm{Tx}}=4\;\mathrm{dBi}$, $L_{\mathrm{Tx}}=4\;\mathrm{dB}$, $G_{\mathrm{Rx}}=4\;\mathrm{dBi}$ and $L_{\mathrm{Rx}}=2\;\mathrm{dB}$. The distant dependant FSPL was calculated according to Equation (2) using the radio channel center frequency 5,9GHz. The deviation between theoretical and measured RSS can be explained by the presence of non-modelled connection losses or imprecise vendor specifications (e.g., on antenna gain). Additionally, an ideal FSPL is assumed which is not entirely met in real-world scenarios. Especially given shadowing, NLoS conditions or multipath caused by vegetation (see Figure 5) or other propagation phenomena. Additionally, Figure 8b shows the absolute frequency of measured RSSI, indicating several accumulation points. This effect is intuitive as a repetitive drive along a circular trajectory was performed. The occurring variations in RSSI can also be reasoned with the aforementioned possible obstacle influence as well as the only partially available antenna patterns. Especially, a lack of information for antenna gain given different elevation angles is crucial, since rover and base are not located in a 0° elevation planar setup to each other.

In summary it can be concluded that for a limited local area given minor obstacles, hindering LoS reception, DSRC still provides a reliable communication link, suitable for broadcasting GNSS correction data.

### 3.3. GNSS Positioning Performance

Concerning the GNSS performance, the accuracy is measured by the spatial discrepancy between a position estimate and the reference position, as described in Section 2.1.2. The horizontal position deviation is used as main performance parameter and accordingly the RMSE of this measure is the performance indicator as defined in Equation (15). All error values and their corresponding statistical parameters are presented in Table 3.

#### 3.3.1. Single Positioning

**Figure 9:**
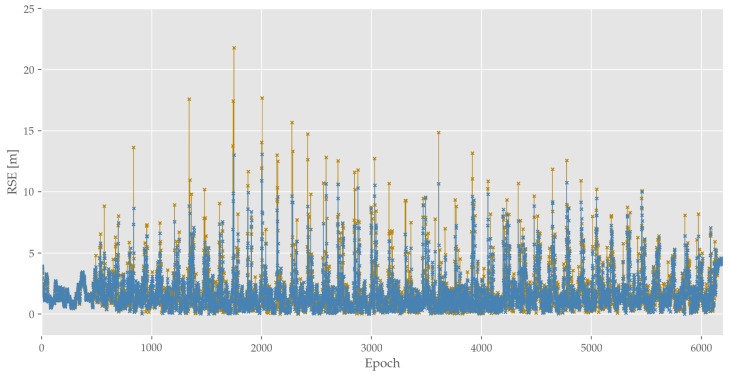
Depiction of epoch-wise RSE for SP: LSE (yellow) and EKF (blue).

At first, the positioning accuracy of stand-alone SP-LSE and SP-EKF is examined. An epoch-wise visualization of SP-LSE and SP-EKF RSE performance is shown in Figure 9. As indicated by this figure, the non-cooperative approaches reach a reasonable accuracy with a RMSE of $Q_{\mathrm{RMSE}}=1.8\;m$. Putting the corresponding variances in perspective, reveals a SP-EKF superiority of 22% when compared to SP-LSE. However, the overall averaged performance of both methods in absolute numbers is very comparable. Additionally, observing the given error quantity frequency also reveals a cyclic appearance of positioning outliers, which will further be discussed in Section 3.4. Comparing the two SP approaches, there are fewer outliers or rather less significant outliers in the Kalman-based approach, which can be explained due to the prediction-correction structure and parameterization, not allowing for unreasonable trajectory jumps. For instance, this can be quantitatively described by eyeing maximum outliers, which are given with:

$${\underset{\max}{Q}}^{\mathrm{SP}-\mathrm{LSE}}=21.8\;m\;\mathrm{and}\;{\underset{\max}{Q}}^{\mathrm{SP}-\mathrm{EKF}}=13.1\;m$$

This is also backed up by further quantitative analysis in Table 3, as the RSE for 3σ-Quantile for SP-LSE with $Q_{\mathrm{RSE}}=8.8\;m$ is comparably high to the EKF’s of $Q_{\mathrm{RSE}}=7.6\;m$, while resulting in the same average.

**Figure 10:**
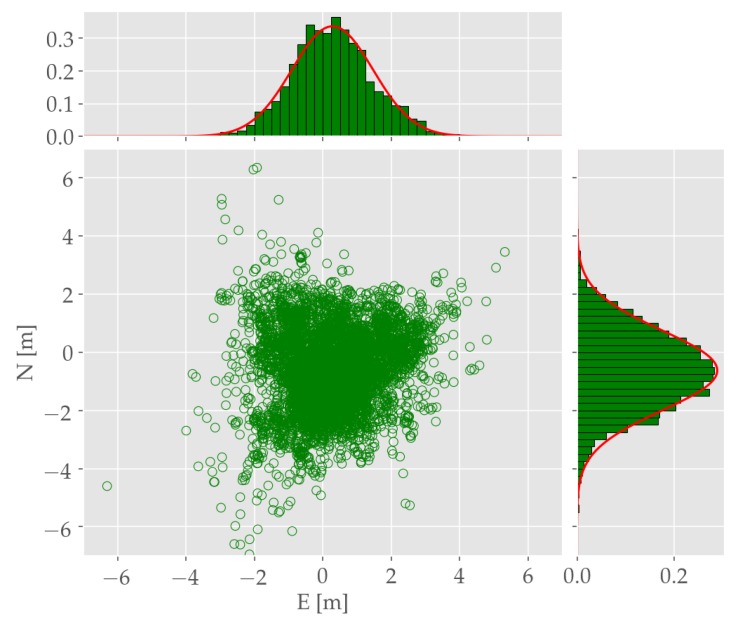
Horizontal position estimates and error distribution of base station single positioning.

In addition to the SP rover performance, base accuracy is also discussed, since its performance influences the overall positioning result in CP approaches. As one can discern from Figure 10, the horizontal RSE of the base station is a lot less influenced by outliers possibly caused by satellite shadowing or NLoS reception due to its exposed position. An examination of the east and north coordinate is shown in the provided histograms in Figure 10. The horizontal error components of the LSE processed base station measurements are normally distributed, which prompts towards a normal distribution of the unmodelled residual error. The estimated distributions are: $e_{b}∼N\left(0.28,1.19\right)$ and $n_{b}∼N\left(-0.62,1.38\right)$. This reveals two conclusions:

1. In accordance to the assumption that the base station’s observations must not be strained by multipath influences in order to serve as unbiased reference.
2. As indicated in Table 2 variance propagation in dependence on the amount of differencing steps is performed. Therefore, base variance is highly influential on CP accuracy performance.

#### 3.3.2. Cooperative Positioning

**Figure 11:**
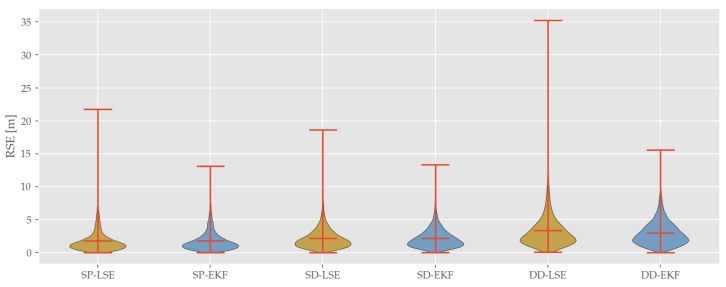
Comparison of error distributions of all applied positioning approaches.

The results of the CP methods in general reveal similar qualitative effects to the already discussed SP approaches (Figure 9). Even though numbers vary, both SD and DD also show cyclically recurring positional peaks. In addition, EKF approaches beat LSE approaches on overall accuracy, outlier mitigation and variance for SD and DD. Comparing the overall performance of the two differencing approaches reveals the downside of DD. As can be seen in Figure 11, which contains violin plots for all presented approaches (indicating median, whiskers and estimated error distribution which are estimated by applying Kernel Density Estimation of the RSE), and in Table 3 DD performs significantly worse for all statistical measures when compared to SP and SD.

In summary, two main tendencies are observable:

1. EKF yield lower RMSE as well as lower outliers compared to their respective LSE counterpart
2. Differencing inducts a higher mean and median error as well as a higher error variance

The fact that differencing induced an higher error seems counter-intuitive in the first place. In contrary to that assumption, it was shown that variance propagation of both base and rover station are essential for the resulting CP accuracy. Apparently, the quality of a low-cost base station in connection with a low-cost antenna does not provide good enough PR correction to achieve higher accuracies than stand-alone positioning. In addition to that, the influence of unmodelled stochastic errors also increase on each differencing step.

**Figure 12:**
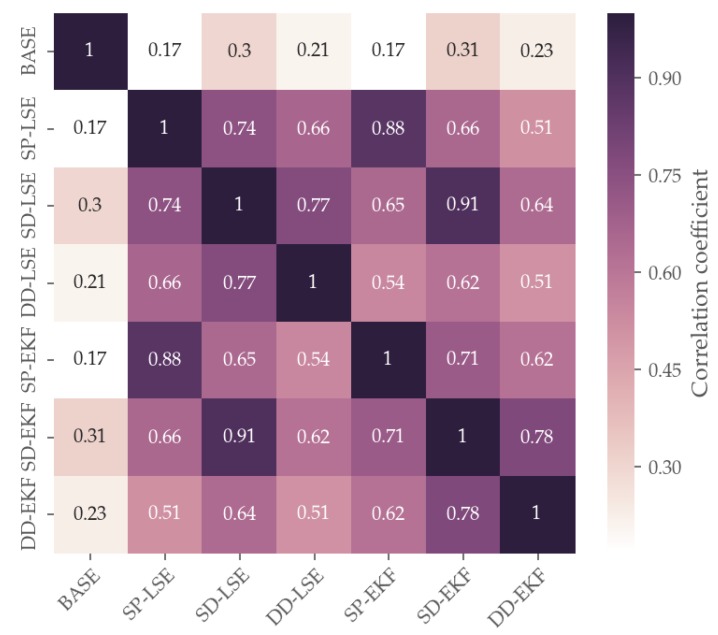
Correlation analysis of 2D-RSE for all introduced positioning methods.

In order to further investigate the relationship between rover and base, additional analysis were performed. With this in mind, the first fact to study in detail is the correlation of the error regimes of different algorithms. A possible way of comparison is to take the epoch-by-epoch horizontal error, which will render a time correlation in the errors visible. Examining Figure 12, it becomes apparent that the base’s error is almost uncorrelated to the rover’s SP error with an correlation coefficient of 0.17 for BASE and SP-LSE, which indicates a weak correlation of pseudorange errors in the measurement domain as well. As both receivers are using the same hardware and same error models for atmospheric refraction, this suggests that the main error causes can be found in the direct vicinity of the receiver as a consequence of multipath and NLoS effects.

**Figure 13:**
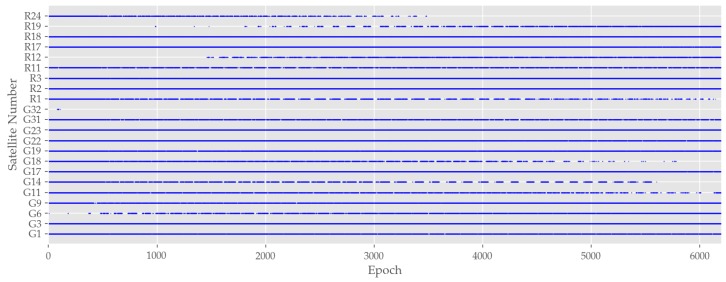
Observable satellites during the measurement run. Dashed lines indicate that the corresponding satellite was only visible at certain times.

The presumption of NLoS effects can be further examined by a satellite-receiver visibility analysis. Looking at Figure 13, one can see in a glance that there are periodic reception outages for several satellites. The intermittency in the satellite’s visibility suggests that the outages occur always on the same segments of the test trajectory. The assumption of cyclic outliers is also backed by the analysis of satellites in view in Figure 13, which reveal a returning outage of signal reception of several satellites, including, for example, *G14, G18* and *R1*. A closer look at Figure 9 reveals an interval of positional outliers approximately every 120 to 140 epochs. A similar occurrence can be observed at reception outage of just mentioned satellites. This can be explained by the testbed’s geometry as well as vehicle velocity during the testrun. The testbed’s center line has an approximate length of l=250 m. The testrun was performed with an average speed of about v=15 km/h, which equals about 4 m/s. This results in a lap time of approximately $t_{\mathrm{lap}}=60\;s$. Considering the already mentioned measurement rate of 2 Hz, a revisiting of neuralgic points can be identified.

### 3.4. Visibility and NLoS Analysis

As previously mentioned, there are recurring outliers in the position error. As we conducted our measurement by repeatedly driving on the same trajectory, the conclusion is obvious that some property of the environment may affect the position accuracy. As discussed in Section 2.2, the unmodelled residual error is increased in cooperative positioning approaches due to covariance propagation. As there are several tall buildings in the vicinity of the test track, a visibility and NLoS analysis is conducted in order to further examine this possible error source.

**Figure 14:**
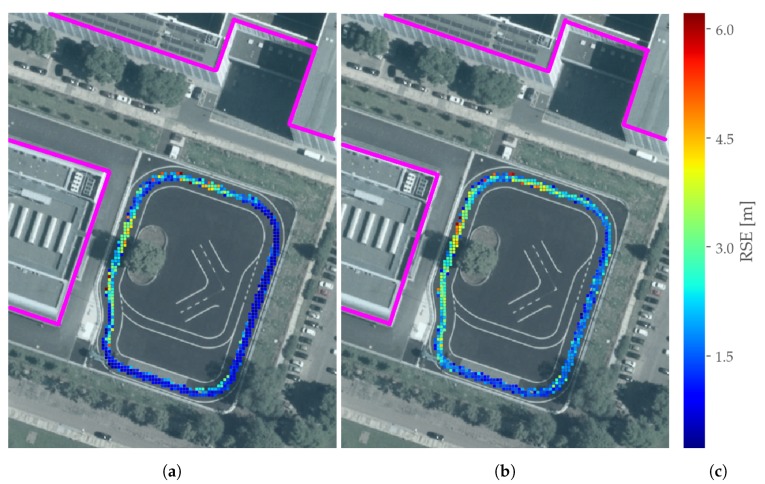
Spatial distribution of horizontal positioning errors in an equidistant grids with an edge length of 1m: (**a**) SP-LSE and (**b**) SD-LSE with associated RSE quantities in (**c**). Edges of buildings higher than 10 m are marked in pink.

Figure 14 depicts the horizontal error in the estimated positions for each part of the test trajectory that the vehicle’s position was measured. The colorbar was created by mapping those positions to a equidistant grid of 1 m. At the end, each grid cell displays the RMSE over the entire test drive for their respective locations. Apparently there are two main areas in which the error value are higher than elsewhere, which are also close to the main buildings next to the test track. In contradiction, in the south-east part of the track bordering to parking lots only low errors were experienced.

**Figure 15:**
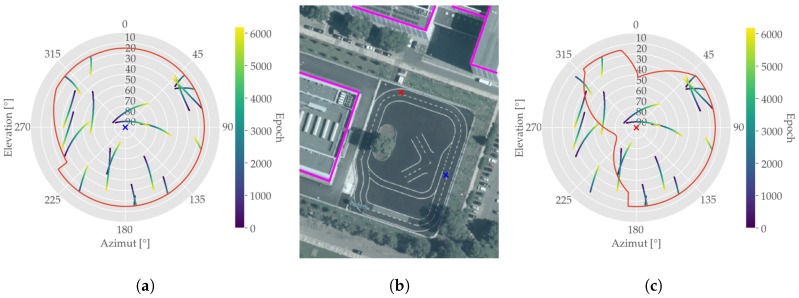
Comparison of sky occupancy for two points on the measuring trajectory. The red line in the skyplots depicts the visual horizon line with a general elevation mask of 15°. (**a**) Next to buildings indicating a limited open sky field of view; (**b**) Location for both examination points; (**c**) Open sky conditions.

In order to examine the assumption of multipath influence further, two representative points were chosen and a visibility horizon was calculated. For integrating the buildings, a digital 3D model of the area was used. The resulting skyplots for both points are shown in Figure 15. From the left subplot its observable that at the point next to the buildings (red marker) the visual horizon is retrenched especially in the western azimuth section. In contradiction, the conditions for GNSS signal reception on the eastern part of the test track are almost unconstrained, as shown in the right subplot of Figure 15 (blue marker).

As for low positioning accuracy and limited sky view coincidence, this suggests a causality caused by NLoS and signal shadowing. This is backed by the literature as explained in Section 2.2. However, as no explicit multipath modelling was performed, this may still be only considered as an assumption, yet very likely to be true given the circumstances. Furthermore, the signal outages can be explained with this modelling. Based on these findings, countermeasures for mitigating NLoS reception can be undertaken in order to improve GNSS positioning performance. This is discussed in the following Section 4.

## 4. Conclusions

Vehicular communication and HAD are major emerging trends in the field of modern multi-modal mobility. Both are tightly intermeshed as the advance of ADAS and HAD functions require environmental perception that exceeds their own on-board sensors. Moreover, knowledge about the vehicle’s current position is crucial for both safety-critical ADAS and HAD, as well as for traffic management, LCA and consumer applications. In addition to that, a number of communication-aided LBS arose, also depending on an accurate user position estimation. For plenty of these applications, the accuracy performance of stand-alone GNSS, which is typically used for global positioning, do not meet the necessary requirements.

As a solution to this, cooperative positioning (CP) algorithms like DGNSS and RTK are suggested in order to improve the positioning accuracy. These approaches have already been extensively evaluated for various applications. One of the cornerstones of such a CP scheme is the communication of correction data from the fixed base station to the vehicle. We evaluated the quality and adaptability of an IEEE 802.11p airlink for dissemination CP correction data. Regarding this communication link’s performance we obtained a package success rate of 100%, which means the radio link works reliably within the range of 80 m even when direct LOS is obstructed by vegetation.

Concerning the position estimation, we have implemented LSE and EKF based single positioning, single differencing and double differencing filters. For all three methods, we have shown both EKF and LSE implementations perform similarly in average numbers (RMSE and MAE). Further on, it was revealed that EKF variants are generally more robust to outliers which is observable in a lower error variance (e.g., SP-LSE: $\sigma_{\mathrm{RMSE}}^{2}=3.1\;m$ and SP-EKF: $\sigma_{\mathrm{RMSE}}^{2}=2.4\;m$) as well as significantly smaller 3σ error quantiles.

It was shown that the necessary performance demands for SCA and LCA in terms of accuracy (0.7 to 1.1 m) are not met by only using low-cost GNSS based single point positioning or DSRC enhanced CP approaches (SP-EKF: $Q_{\mathrm{MAE}}=1.4\;m$ and SD-EKF: $Q_{\mathrm{MAE}}=1.8\;m$) in the given scenario. As suggested in Section 2.1.2, we found NLoS effects were a main error source in dense urban environments. Cooperative positioning algorithms are especially affected by this, as uncorrelated errors between base and rover are amplified due to the differencing approach, which can be observed in comparing RMSE, for example, SP-LSE: $Q_{\mathrm{RMSE}}=1.8\;m$, SD-LSE: $Q_{\mathrm{RMSE}}=2.2\;m$ and DD-LSE: $Q_{\mathrm{RMSE}}=3.3\;m$. For investigating this, a skyview analysis was performed. By calculating a virtual horizon using a 3D building model, NLoS satellites can be identified. It was shown that parts of the test trajectory with limited sky view coincidence with high horizontal errors in the position estimates. Overall it is concluded that cooperative positioning approaches are often being challenged by these NLoS effects and are therefore less reliable than single positioning approaches with error modelling.

Based on these findings, new ways of multipath detection and error mitigation need to be researched. Exploiting the idea of 3D environment models further, they can be used in this sense by identifying and NLoS satellites and adaptively weighting such observables. Another possibility is the incorporation distance estimations based on RSSI measurements of the IEEE 802.11p airlink in the position estimation filter as a transponder-based augmentation approach or the use of information fusion with additional vehicle dynamic sensors, like accelerations, travel speed and heading, can be performed to accurately estimate vehicle trajectories. Over all, the research of NLoS resistant augmentation technologies for GNSS based positioning is a precondition for future connected urban automotive mobility systems.

## Figures and Tables

**Table 1:** Overview of structure and components of the Extended Kalman Filter.

Filter Step	Equations	Symbols	Dim.	Description
Initialization		x	n×1	State vector
		$\Sigma_{P}$	n×n	State covariance matrix
		$\Sigma_{Q}$	n×n	Process noise matrix
		$\Sigma_{R}$	m×n	Measurement noise matrix
Prediction	${\hat{x}}_{k|k-1}=f_{F}\left({\hat{x}}_{k-1}\right)$	$f_{F}$	n×n	State transition function
	${\hat{\Sigma }}_{P_{k|k-1}}=J_{F}{\hat{\Sigma }}_{P_{k-1|k-1}}J_{F}^{⊺}+\Sigma_{Q_{k}}$	$J_{F}$	n×n	State transition Jacobi matrix
Correction	$y_{k}=z_{k}-f_{H}\left({\hat{x}}_{k|k-1}\right)$	y z	m×1 m×1	Innovation vector Measurement vector
	$K_{k}={\hat{\Sigma }}_{P_{k|k-1}}J_{H}^{⊺}\left(J_{H}{\hat{\Sigma }}_{P_{k|k-1}}J_{H}^{⊺}+\Sigma_{R_{k}}^{-1}\right)$	$f_{H}$ $J_{H}$	m×n m×n	Measurement function Measurement Jacobi matrix
	${\hat{\Sigma }}_{P_{k}}=\left(I-K_{k}J_{H}\right){\hat{\Sigma }}_{P_{k|k-1}}$	K	m×n	Kalman gain
	${\hat{x}}_{k|k}={\hat{x}}_{k|k-1}+K_{k}y_{k}$	I	n×n	Identity matrix

**Table 2:** Comparison of observation differencing approaches for GNSS PR measurements.

	SP	SD	DD
**Differences**	**None**	**Receiver**	**Receiver Satellite**
Atmospheric errors	existent	reduced	strongly reduced
Satellite clock bias	existent	eliminated	eliminated
Receiver clock bias	existent	existent	eliminated
Stochastic errors	existent	existent	existent
Error covariance	$\sigma^{2}$	$2\sigma^{2}$	$4\sigma^{2}$

**Table 3:** Quantitative evaluation of all positioning methods.

Const.	$\bar{\mathrm{GDOP}}=1.6\;\;\bar{\mathrm{PDOP}}=1.4\;\;\bar{\mathrm{HDOP}}=1.1\;\;\bar{\mathrm{VDOP}}=0.9$															
Metric		RMSE							MAE							
Measure	Q	$\sigma^{2}$	Quantile[m]			Percentile[m]			Q	$\sigma^{2}$	Quantile[m]			Percentile[m]		
	[m]	${\left[m\right]}^{2}$	σ	2σ	3σ	25	50	75	[m]	${\left[m\right]}^{2}$	σ	2σ	3σ	25	50	75
BASE	1.7	0.8	2.0	3.3	4.4	1.1	1.6	2.2	1.5	1.5	1.9	3.9	5.5	0.6	1.2	2.1
SP-LSE	1.8	3.1	1.8	5.3	8.8	0.8	1.3	2.1	1.4	4.5	1.3	4.9	10.9	0.4	0.8	1.6
SP-EKF	1.8	2.4	1.9	5.0	7.6	0.8	1.4	2.2	1.4	3.3	1.4	4.9	9.6	0.4	0.9	1.7
SD-LSE	2.2	2.6	2.4	5.1	8.1	1.1	1.8	2.8	1.8	3.8	2.0	5.2	9.6	0.6	1.4	2.4
SD-EKF	2.2	2.2	2.5	5.0	7.5	1.1	1.8	2.9	1.8	3.2	2.1	5.3	8.9	0.7	1.4	2.4
DD-LSE	3.3	8.3	3.6	8.3	15.0	1.6	2.6	4.1	3.1	14.3	3.3	9.4	18.8	1.0	2.1	3.9
DD-EKF	3.0	3.6	3.5	6.5	9.2	1.6	2.6	3.9	2.6	6.1	3.0	7.4	11.7	0.9	2.0	3.6
